# Response of Antarctic cryoconite microbial communities to light

**DOI:** 10.1093/femsec/fiw076

**Published:** 2016-04-18

**Authors:** Elizabeth A. Bagshaw, Jemma L. Wadham, Martyn Tranter, Rupert Perkins, Alistair Morgan, Christopher J. Williamson, Andrew G. Fountain, Sean Fitzsimons, Ashley Dubnick

**Affiliations:** 1 School of Earth and Ocean Sciences, Cardiff University, Cardiff CF10 3AT, UK; 2 Bristol Glaciology Centre, School of Geographical Sciences, University of Bristol, BS8 1SS, UK; 3 Departments of Geology and Geography, Portland State University, Portland, Oregon OR 97201, USA; 4 Department of Geography, University of Otago, Dunedin, PO Box 56, New Zealand; 5 Earth and Atmospheric Sciences, University of Alberta, Edmonton, Alberta, TG6 2E3, Canada

**Keywords:** cryoconite, glaciers, PAR, photophysiology

## Abstract

Microbial communities on polar glacier surfaces are found dispersed on the ice surface, or concentrated in cryoconite holes and cryolakes, which are accumulations of debris covered by a layer of ice for some or all of the year. The ice lid limits the penetration of photosynthetically available radiation (PAR) to the sediment layer, since the ice attenuates up to 99% of incoming radiation. This suite of field and laboratory experiments demonstrates that PAR is an important control on primary production in cryoconite and cryolake ecosystems. Increased light intensity increased efficiency of primary production in controlled laboratory incubations of debris from the surface of Joyce Glacier, McMurdo Dry Valleys, Antarctica. However, when light intensity was increased to levels near that received on the ice surface, without the protection of an ice lid, efficiency decreased and measurements of photophysiology showed that the communities suffered light stress. The communities are therefore well adapted to low light levels. Comparison with Arctic cryoconite communities, which are typically not covered by an ice lid for the majority of the ablation season, showed that these organisms were also stressed by high light, so they must employ strategies to protect against photodamage.

## Abstract (precis)

Microbial communities in cryoconite holes and cryolakes on glaciers in Antarctica are adapted to low light levels and exhibit stress when exposed to high light.

## INTRODUCTION

Glacier surface microbial communities are significant contributor to local and regional carbon cycles (Anesio and Laybourn-Parry 2012). They include snow and ice algal communities which directly colonize the ice surface (Yallop *et al.*^2012^; Lutz *et al.*^2014^), and accumulations of debris known as cryoconite holes and cryolakes. These are formed when debris deposited on the ice surface, by wind transport or avalanching from valley sidewalls, melts down into the ice, forming a pool of water (Hodson *et al.*^2008^). In the McMurdo Dry Valleys, Antarctica, low temperatures promote the formation of an ice lid which refreezes over the debris, causing the cryoconite debris and water pocket to become sealed off from the atmosphere and surrounding drainage system (Fountain *et al.*^2004^). The cryoconite holes are generally stable, persist for several ablation seasons and can remain isolated for years at a time (Tranter *et al.*^2004^; Bagshaw *et al.*^2007^).

Larger accumulations of debris also form on the glacier surfaces, as a result of redistribution of cryoconite debris via supraglacial meltwater flows. These form in depressions on the ice surface and store meltwater for extended periods during its transition through the supraglacial drainage system (Fountain *et al.*^2004^). They are known as ‘cryolakes’, and generally range in scale from 2 to 15 m in width (Bagshaw *et al.*^2010^). They differ from Arctic supraglacial lakes by their long-term persistence, the presence of an ice lid and the thick layer of debris which forms at their base. Water depths range from 5 to 50 cm, and the sediment layer is typically several centimeters deep.

Cryolakes and cryoconite holes host a variety of microorganisms, including cyanobacteria, tardigrades and rotifers (Christner, Kvitko and Reeve 2003), and are likely inoculated by wind-blown fragments of algal mats and desiccated microorganisms (Nkem *et al.*^2006^). They provide a sheltered habitat on the ice surface where the microorganisms have access to liquid water, dissolved nutrient and sunlight (Tranter *et al.*^2004^; Hodson *et al.*^2008^; Anesio *et al.*^2009^), although the ice lids sometimes afford a high degree of shading. The organic carbon and nutrient stored in these glacier surface habitats can have wide-ranging impacts on downstream ecosystems. It has been suggested that nutrients flushed from glacier surfaces can support biological processes in glacier forefields and ice-covered lakes (Foreman, Wolf and Priscu 2004; Hood *et al.*^2009^; Singer *et al.*^2012^). This is particularly important in the nutrient-poor McMurdo Dry Valleys of Antarctica, where episodic warming events can cause widespread flushing of the glacier surface. The consequent elevated export of nutrient from the glacier surface promotes enhanced primary production in the downstream ice-covered lakes (Foreman, Wolf and Priscu 2004; Bagshaw *et al.*^2013^).

Cryoconite holes and cryolakes accumulate carbon and bioavailable nutrients over time. New autochthonous organic carbon accumulates when photosynthesis or production of organic matter (P) is greater than the respiration or consumption of organic matter (R). The production of autochthonous organic carbon in and on glacier surfaces has been investigated worldwide, although most studies have been conducted in the Northern Hemisphere and have focused on cryoconite holes (Laybourn-Parry, Tranter and Hodson 2012). There has been debate in the literature as to how and when the microbial communities reside in a state of net production of organic matter (net P) or net consumption (net R). In this paper, we determine the circumstances in which the systems are in the state of net production, and investigate the role of the ice lids in regulating carbon cycling. We use a combination of *in situ* measurements of environmental and physical parameters (light, temperature, electrical conductivity (EC) of meltwaters) and controlled laboratory incubations to measure changes in oxygen concentration over time, as a proxy for primary production (Bagshaw *et al.*2011a). We also employ pulsed amplitude modulated (PAM) fluorometry to assess changes in photophysiology and photosynthetic efficiency during the course of long-term incubation experiments. Samples from Antarctica were compared with those from Greenland, where the cryoconite communities are not covered by an ice lid during the peak of the ablation season.

## METHODS

### Field measurements

**Figure 1.**
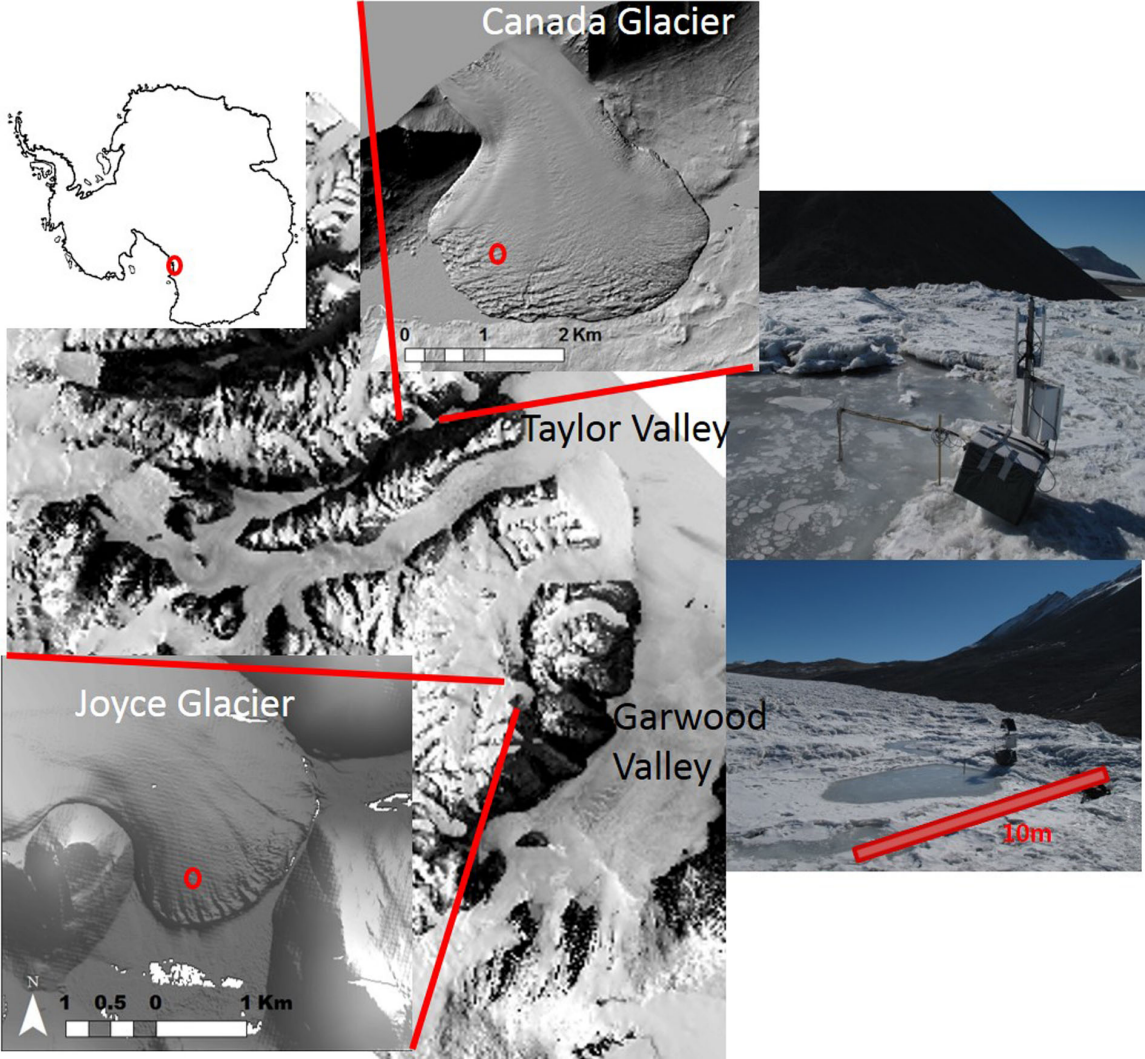
Location of Joyce Glacier, Garwood Valley, McMurdo Dry Valleys, Antarctica, where temperature, DO and EC probes were installed in a cryolake in January 2010. The map also shows where samples were collected for laboratory incubations from a cryoconite hole on Canada Glacier, Taylor Valley, Antarctica.

We used *in situ* monitoring probes to measure physical and biogeochemical conditions in cryolakes and cryoconite holes on the surface of two glaciers in Antarctica. Dissolved oxygen (DO) optodes (PreSens Fibox 3), EC and temperature probes (the latter two custom built, 1.5 cm diameter; Bagshaw *et al*. 2011a) were installed in a cryolake on Joyce Glacier (78.0243°S, 163.7788°E) for 25 days in January 2010 (Fig. 1). A small (<5 cm) diameter hole was drilled into the 15 cm thick ice lid of the 6 m x 2 m cryolake. The monitoring probes were inserted into the hole and allowed to freeze in place, some 8 cm above the bed. Apogee quantum sensors were also installed to simultaneously monitor PAR (photosynthetically available radiation) on both the ice surface and immediately below the ice lid. Manufacturer's estimates of precision and accuracy are <5%. The sensors were controlled by Campbell Scientific CR10X data loggers, powered by 12 V batteries that were trickle charged by solar panels. Measurements were taken every 30 s and logged every 15 min. All sensors remained *in situ* for up to 1 month.

Estimates of PAR attenuation under different thicknesses of ice lid in the cryolake were obtained via manual measurements of PAR beneath differing thickness of ice and water, using a hand-held Apogee flux meter and the Apogee quantum sensor. One sensor was held in the cryolake beneath the ice lid and compared with the permanent sensor on the ice surface. Light attenuation in open cryolake water (i.e. lake water with no ice cover) was estimated using the same approach. Ice thickness measurements were taken in contrasting cryolakes located close to the main monitored lake on three different days (12, 14 and 27 January), which covered a wide range of ice thicknesses. Each day was characterized by clear skies, and seasonal fluctuations in PAR at the surface should be accounted for by the calculation of percentage attenuation. Mean ice lid thickness of the Joyce Glacier cryolake was 16 cm (n = 28); mean cryoconite hole ice lid thickness on Canada Glacier (from 89 samples in 2005/6) was 9 cm.

### Long-term laboratory incubation experiments: cryolake debris from Joyce Glacier

Long-term incubations under controlled laboratory conditions were designed to simulate the establishment of a cryolake ecosystem over long timescales, and to study the effect of variations in light intensity. Debris samples from a cryolake on Joyce Glacier were transported frozen to the University of Bristol's LOWTEX facility, and stored frozen at –25°C in the dark for 17 months prior to experimentation. The frozen samples were slightly thawed, in the dark, to allow 25 g to be transferred to glass bottles for incubation, to a depth of 1 cm. The bottles were filled to overflowing with deionized water (∼100 ml) and stopped with gas tight, needle-penetrable bungs. The bottles were crimped and remained sealed for the duration of the experiment. Bottles were divided into light, dark and control (in duplicate). Bottles were immersed in a water bath beneath fan-cooled horticultural lighting (Envirolite) in a water bath within a cold room where the temperature was maintained between 0°C and 1.5°C. Differing levels of shading over the bottles mimicked the ice lids of the cryolakes, and were maintained by covering sections of the water bath with varying quantities of tissue paper. PAR was measured continuously at all four shading conditions (referred to as 0%, 25%, 50% and 75% shading), and temperature was monitored throughout the experiment. PAR generated by the Envirolite rig was 60 μmol m^2^ s^−1^ under non-shaded conditions, and 45, 25 and 15 μmol m^2^ s^−1^ under the 25%, 50% and 75% shades, respectively. This compares with peak values of 1000 μmol m^2^ s^−1^ on the ice surface and 400 μmol m^2^ s^−1^ at the base of a cryoconite hole on Canada Glacier, and peaks of 1200 and 660 μmol m^2^ s^−1^ above and below the ice lid of a cryolake on Joyce Glacier.

DO measurements were conducted using a needle-type optode (PreSens Microx), which can penetrate bottle stoppers while maintaining a gas-tight seal. The needle probe was inserted into the bottles daily for the first month of the experiment, and thereafter every 7–14 days up to 145 days. Needles remained in each bottle for 1 min during measurement. Measurements were logged every 10 s using a Campbell Scientific CR1000 datalogger, which recorded a phase shift of the LED beam proportional to the *in situ* oxygen concentration (Bagshaw *et al.*2011b). The oxygen concentration in each bottle was determined by first taking a mean of the six readings in the individual bottles, and then in duplicate bottles of each type (light/dark/control/degree of shading). Reported error bars show the range of the readings around this mean.

In order to investigate the processes occurring in greater detail, a second incubation experiment was conducted using cryoconite samples from Canada Glacier, McMurdo Dry Valleys, Antarctica (77.62° S, 162.95° E, Fig. 1) and Leverett Glacier, SW Greenland (67.06 °N, 50.17 °W). Debris samples used were already in house at Cardiff University, UK, having been transported, stored and transferred to incubation vessels as in the first experiment. The incubation was performed in a cold room where the water bath was maintained at 4.5°C, under LED light rigs from Dormgrow (USA). The LED lights covered the UV, blue (280–495 nm), red, far-red and white (620–900 nm) parts of the spectrum, to closely mimic the natural radiation received by the organisms at the ice surface. The LED lights provided higher PAR than the Envirolite rigs, so the experiment was conducted at 274 and 75 μmol m^2^ s^−1^. DO measurements were conducted in a similar manner to the first incubation, but instead used a Unisense needle-type optode (Unisense, Denmark).

### Determination of P and R

P (photosynthesis) and R (respiration) as μgC g^−1^ day^−1^ were calculated by Equations 1 and 2 (Telling *et al.*^2010^), where O_2_^P^ is the recorded saturation of oxygen in the light sample (%), O_2_^R^ is the recorded saturation of oxygen in the dark sample (%), O_2_^Blk^ is the oxygen saturation of the blank, O_2_^0°C^ is the oxygen content of water at 0°C (14 mg/l), 32 is the molecular mass of oxygen, 12 is the relative atomic mass of carbon and Hours is the duration of each incubation step in hours. There was no correction for sulfide oxidation, since previous investigation has shown it is insignificant compared with DIC generated by ecosystem productivity (Hodson *et al.*^2010^). Sulfate concentrations ranged from 0.4 to 1.9 ppm. NEP (net ecosystem production) is calculated by the difference between P and R.

(1) \begin{eqnarray*} P = \frac{{\left( {{\rm{O}}_2^P - {\rm{O}}_2^R} \right){\rm{O}}_2^{0^\circ {\rm{C}}} \cdot {\rm{Mas}}{{\rm{s}}_{{\rm{water}}}} \cdot 12}}{{32 \cdot {\rm{Mas}}{{\rm{s}}_{{\rm{sediment}}}} \cdot 100\ \cdot \left( {\frac{{{\rm{Hours}}}}{{24}}} \right)\ }} \\ \nonumber \end{eqnarray*}

(2) \begin{equation*} R = \frac{{\left( {{\rm{O}}_2^R - {\rm{O}}_2^{{\rm{Blk}}}} \right)\ {\rm{O}}_2^{0^\circ {\rm{C}}} \cdot {\rm{Mas}}{{\rm{s}}_{{\rm{water}}}} \cdot 12}}{{32 \cdot {\rm {Mas}}{{\rm{s}}_{{\rm{sediment}}}} \cdot 100 \cdot \left(\frac{\rm {Hours}}{24} \right)}} \end{equation*}

### Fluorescence measurements of photophysiology

PAM fluorescence measurements were carried out in the laboratory using a Water PAM with EDF fiber optic blue light detector/emitter PAM (Walz, Effeltrich, Germany) with a blue measuring light. Blue light was used, rather than the more common red light, since the dominant species in Antarctic cryoconite is cyanobacteria (Porazinska *et al.*^2004^). Rapid light photosynthesis-irradiance curves (RLCs) were constructed following the method of Perkins *et al.* (2006, 2010) using incremental light steps of 30 s duration and a saturating pulse of approximately 8000 μmol m^−2^ s^−1^ PAR (400–700 nm) and pulse width of 600 ms. Data were imported into R (R v.3.0.2; R Core Development Team 2008) software and relative electron transport rate (rETR) was calculated as Equation 3, where *Fq′/Fm′* is the quantum efficiency of PSII calculated by Equation 4, and where *Fm′* is the maximum fluorescence yield and *F′* is the operational fluorescence yield at each incremental light step of the RLC.

(3) \begin{equation*} {\rm{rETR}} = {F'_q}/{F'_m} \times 0.5 \times {\rm{PAR}}\end{equation*}

(4) \begin{equation*}{F'_q}/{F'_m}{\rm{ = }}\left( {Fm'-F'} \right)/Fm' \end{equation*}

For each RLC, iterative solution to the curve was carried out using the model of Eilers and Peeters (1988), with calculation of light curve parameters from significant (*P* < 0.01) fits of the coefficients a, b and c from the model. Parameters calculated were α (light utilization coefficient), E_k_ (light saturation coefficient) and rETR_max_ (maximum rETR). Downregulation of photochemistry in the form of non-photochemical quenching (NPQ) (Perkins *et al.*^2010^) was calculated as in equation 5, where *F_m._*_max_ is the maximum *Fm’* value obtained during the RLC and hence takes into account residual NPQ retained from the previous light dose prior to the start of the RLC (Serôdio *et al.*^2005^).

(5) \begin{equation*} {\rm{NPQ}} = {{\rm{F}}_{{\rm{m max}}}}/{F'_m}-1 \end{equation*}

## RESULTS

**Figure 2.**
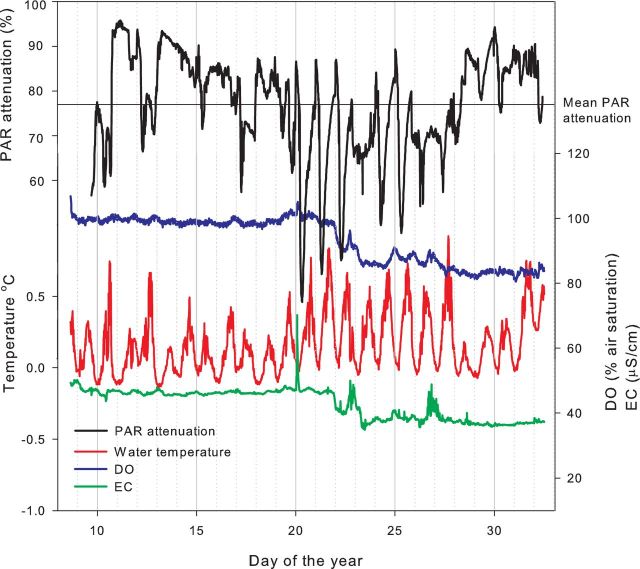
*In situ* measurements of water temperature, EC, DO and PAR attenuation (the fraction of PAR reaching the probe) in a cryolake on Joyce Glacier in January 2010.

The ice thickness of the monitored cryolake ranged from 7 to 30 cm and the water depth from 2 to 28 cm during the experiment. The probes remained frozen in place throughout, although the ice thickness fluctuated. The lake was connected to the surrounding drainage system via a supraglacial stream, but it retained its ice lid throughout the experiment. The *in situ* measurements revealed a complex interplay between physical and biogeochemical processes over the short measurement period (Fig. 2). Changes in PAR at the ice surface caused local heating of the debris surrounding the probe at the base of the cryolake, increasing water temperature and prompting biogeochemical changes. The PAR increase at the cryolake bottom melted the ice above the sediment, releasing solute from the ice crystal matrix, indicated by the spike in EC at DY 20 in Fig. 2. Oxygen concentrations rose from 90% to near 100% air saturation and declined over the following 3 days. There was another peak in EC and oxygen on DY 22, and on DY 26, and then oxygen concentrations stabilized at c. 85% air saturation and EC at c. 38 μS cm^−1^ until the end of the monitoring period.

**Figure 3.**
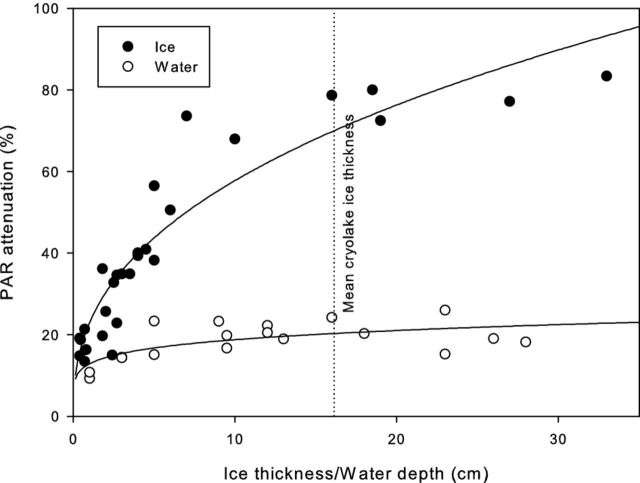
PAR attenuation (surface – subsurface) for ice lids of different thicknesses and with water depth on Joyce Glacier. Regression fit between ice thickness and PAR attenuation is PAR = 22.9 h_i_^0.40^, *r^2^* = 0.87 and water depth and PAR attenuation is PAR = 12.*9* h_w_*^0^*^.14^, *r^2^* = 0.44.

**Figure 4.**
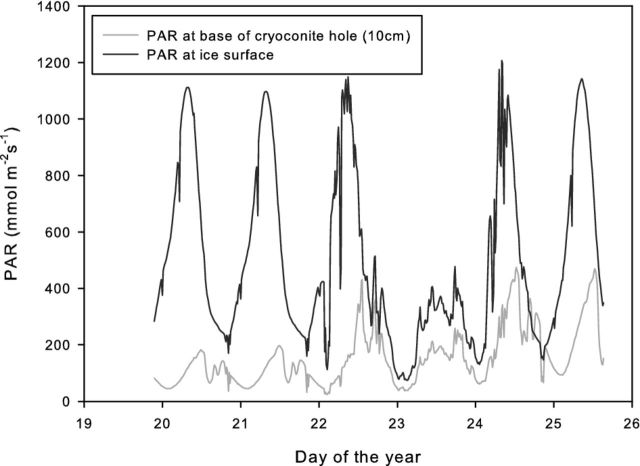
Comparison of PAR receipt at the ice surface of Canada Glacier (January 2010) with that at the bottom of a 10 cm deep, ice-lidded cryoconite hole. Smooth curves are indicative of clear skies. Note that peak PAR at the hole bottom lags that at the surface by 4.5 h and minimum PAR at the bottom either coincides or lags the PAR at the surface.

The mean PAR attenuation, measured by comparing PAR at the surface with that within the monitored cryolake, was 77% (Fig. 2). Experiments on a range of lakes with varying ice lid thickness and water depth showed that the ice was responsible for the majority of the attenuation (Fig. 3). The water layer attenuated approximately 20% of the surface PAR, regardless of the depth, while the ice attenuation was strongly controlled by the ice thickness (Fig. 3). The mean cryolake lid ice thickness was 16 cm (n = 28), which equates to an ice attenuation of 79%, and a water attenuation of 20% (Fig. 3). In cryoconite holes on Canada Glacier, up to 99% of incoming PAR did not reach the base (Fig. 4). Daytime peak values of 1200 μmol m-^2^ s^−1^ on the ice surface of Canada Glacier compared with 475 μmol m^−2^ s^−1^ at the base of the monitored cryoconite hole (overnight minima were 75 and 25, respectively). The mean attenuation in the cryoconite hole was 72% prior to internal melting on 23 January 2010, and 62% thereafter. There was a slight lag between peaks at the ice surface and hole base as a result of glacier sidewall shading: the PAR at the ice surface was derived from an adjacent met station, some 0.5 km from the monitored cryoconite hole, which was shaded slightly earlier than the cryoconite hole site.

### Laboratory experiments

**Figure 5.**
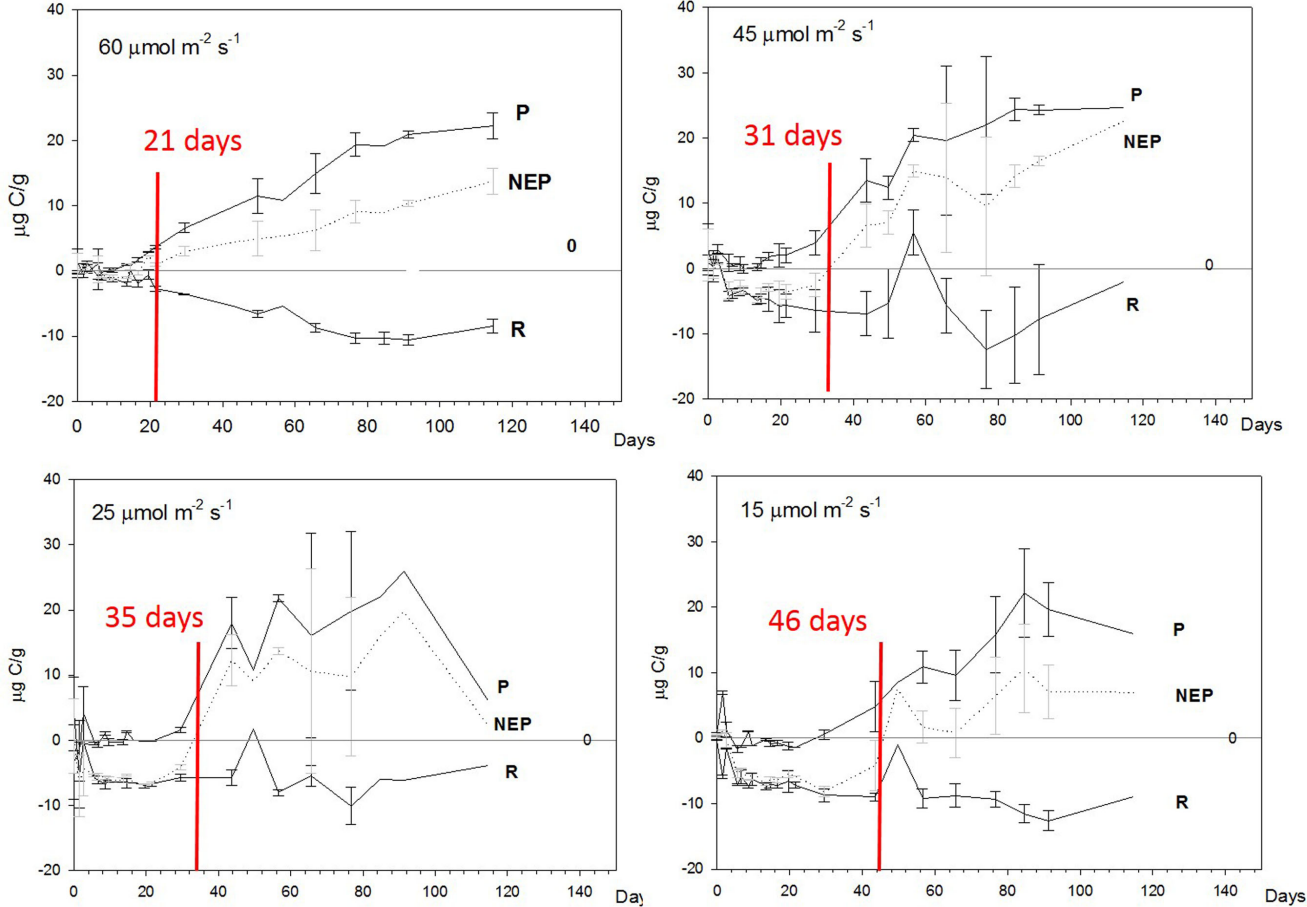
Long-term controlled laboratory incubations of cryolake debris from Joyce Glacier under varying light intensity. The incubation was illuminated by horticultural lighting (Envirolite) and kept in cold room, in a water bath maintained at 0.1°C. The time taken for the community to reach a state of net P (NEP > 0) increased with decreasing light intensity.

The light attenuation data were used to simulate controlled primary production experiments in the lab. In the first experiment, the time taken for the system to reach net P (where P > R, Equations 1 and 2) varied according to the degree of shading (Fig. 5). Where light intensity was higher, the microbial community reached a state of net P more rapidly. The gross quantity of C fixed (Equation 1) in the light bottles peaked at 22.1, 24.6, 25.9 and 22.7 μg C g^−1^ in the 60, 45, 25 and 15 μmol m^2^ s^−1^ experiments, respectively.

**Figure 6.**
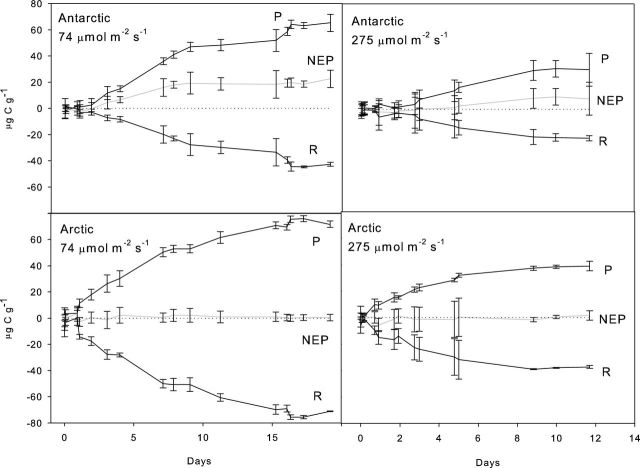
Long-term controlled laboratory incubation of cryoconite debris from Canada Glacier (Antarctic) and Leverett Glacier, Greenland (Arctic) at low (74 μmol m^2^ s^−1^) and high (275 μmol m^2^ s^−1^) light intensity. The incubation was illuminated by LED lighting (Dormgrow) and kept in cold room, in a water bath maintained at 5°C. Production was higher at low light intensity, and the Antarctic community reached a state of net P more rapidly. In the Arctic incubations, P and R remained broadly in balance under both light levels, although activity was higher at low light.

The experiment was repeated at higher light intensities of 74 and 275 μmol m^2^ s^−1^ (Fig. 6). These values were comparable to the peak and minimum concentrations of PAR penetrating to the base of an Antarctic cryoconite hole (Fig. 4). During this experiment, the water bath temperature was higher (4.5°C), so the time taken for the system to reach net P cannot be directly compared with the previous experiment. The gross quantity of C fixed in the light bottles was 30 and 65 μg C g^−1^ in the 275 and 74 μmol m^2^ s^−1^ experiments, respectively. The time taken for the system to reach net P was much faster, likely as a combined result of higher light intensity and higher temperatures. The incubation at 275 μmol m^2^ s^−1^ reached net P after 4 days, whereas the 74 μmol m^2^ s^−1^ incubation took just 1.5 days. This suggests that the higher light intensity may actually inhibit the microbial community.

To investigate whether increasing light intensity could inhibit photosynthetic activity of the phototrophs within the community, we repeated the experiment using cryoconite from an Arctic glacier, Leverett Glacier, SW Greenland and undertook PAM fluorometry measurements throughout the incubation. Cryoconite from the Arctic glacier (67 °N) had been exposed to light for longer periods than the Antarctic glacier (77° S). The Arctic cryoconite was also not covered by an ice lid, so was exposed to high light intensity over the whole summer.

**Figure 7.**
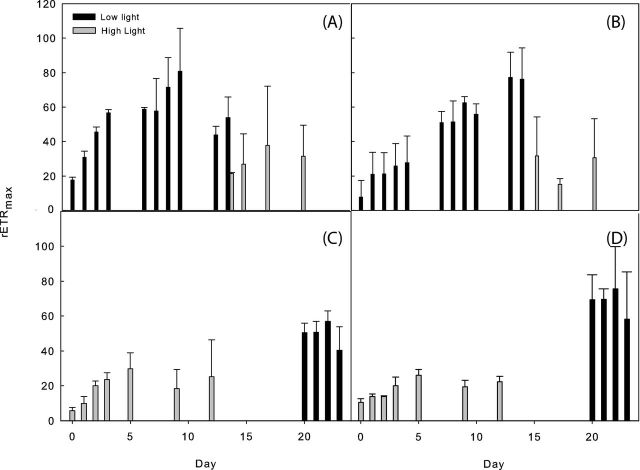
Maximum relative electron transport rate (rETR_max_, a measure of photosynthetic efficiency) during Antarctic (**A** and **C**) and Arctic (**B** and **D**) cryoconite incubations moved between low (74 μmol m^2^ s^−1^) and high (275 μmol m^2^ s^−1^) light conditions. All incubations were more efficient under low light.

The Arctic incubations behaved very differently compared to the Antarctic experiments, in agreement with previous observations in the literature (Hodson *et al.*^2010^; Bagshaw *et al.*2011a). P and R were typically in balance throughout the experiment at both light intensities (Fig. 6), although activity in both light and dark bottles was much higher at 74 μmol m^2^ s^−1^ and there was a very low net P (2 μg C g^−1^) from day 4 onwards. The maximum relative electron transport rate (rETR_max_) shows the rate of electrons pumped through the photosynthetic chain and is hence a measure of photosynthetic efficiency. Fluorescence measurements of rETR_max_ (Fig. 7) indicated that both Antarctic and Arctic samples were better acclimated to low light (74 μmol m^−2^ s^−1^ PAR) compared to high light (276 μmol m^−2^ s^−1^ PAR). Although data were variable between samples (indicated by the high error bars), on transfer from low light to high light (Fig. 7A and B) photoinhibition was observed as a suppression of rETR_max_, suggesting light induced stress, with no acclimation by the end of the experimental period. Importantly, the reverse was seen for transfer from high light to low light, indicating that samples did not acclimate to the higher light level, but showed recovery once transferred into low light (Fig. 7C and D). Data for the coefficient of light use efficiency (α) followed the same pattern as rETR_max_, with correlations of r = 0.76, 0.77, 0.87 and 0.85 (all *P* < 0.01) for Antarctic high to low light, Antarctic low to high light, Arctic high to low light and Arctic low to high light experiments, respectively. Values of α ranged between 0.04 and 0.11 relative units. The light saturation coefficient also followed the same patterns as for rETR_max_ and α, with values ranging between 460 and 660 μmol m^−2^ s^−1^ PAR.

Downregulation in the form of NPQ is a mechanism employed by photosynthetic organisms to protect against photodamage (Ting and Owens 1993). Increasing NPQ induction was apparent as a function of increasing PAR across all light curves of both Arctic and Antarctic samples, in both experiments, either with transfer from high to low or low to high light. Importantly, however, NPQ did not saturate during any rapid light curves, indicating that although photoregulation via NPQ was induced, the highest light dose provided (1944 μmol m^−2^ s^−1^ PAR) was not sufficient to saturate NPQ and cause photoinhibition/photodamage. When Arctic communities were transferred from low light (74 μmol m^−2^ s^−1^) to high light (275 μmol m^−2^ s^−1^), no obvious pattern in NPQ was observed, with an average value of NPQ induced by the end of the rapid light curve (PAR of 1944 μmol m^−2^ s^−1^ PAR) of 0.58 ± 0.012 (mean ± SE). This demonstrated a greater induction of NPQ compared to Antarctic cultures, which had an NPQ of 0.11 ± 0.001 at this light level. Interestingly, Antarctic communities showed a significant linear increase (r^2^ = 0.66, *P* < 0.01) in final NPQ at the end of the light curve, prior to a decline in NPQ to 0.066 when transferred to high light. For samples transferred from high light to low light, there were no significant patterns over the experimental period and no significant difference between Arctic and Antarctic communities, with mean NPQ at the end of the RLC of 0.038 ± 0.0088 and 0.054 ± 0.014, respectively.

## DISCUSSION

Antarctic cryolake and cryoconite hole systems differ from their Arctic counterparts in several key ways. First, the melt season is shorter by 20–40 days; second, they are ice covered, which partially shades the sediment layer; third, the ice cover may hydrologically isolate the hole from surface melt, closing the systems; and fourth, the microbial community may differ (Cameron, Hodson and Osborn 2012), with some samples including small animals which could alter community respiration (Zawierucha *et al.*^2015^). These external controls are important in interpreting ecosystem productivity. For example, freeze-thaw can significantly affect biogeochemical conditions by forcing oxygen in and out of solution and freeze-concentrating solutes in the water (Bagshaw *et al*. 2011a). This can result in spikes in EC and DO (Fig. 4) associated with physical environmental changes rather than biological activity. Our *in situ* monitoring demonstrates the importance of physical changes in controlling the biogeochemical environment within cryoconite and cryolake ecosystems. In order to understand the biological response to these physical changes, we use closed bottle incubations in the laboratory where external physical conditions can be closely controlled.

Light and temperature are important controls on glacial microbial community production (Stibal *et al.*^2012^). In glacier surface ecosystems which contain meltwater, temperatures are fairly constant at just above freezing, and research has demonstrated that bacterial communities are well adapted to cope with these temperatures (Singh, Singh and Dhakephalkar 2014). There has been comparatively little focus on the mechanisms by which these communities respond to intense UV radiation received at the ice surface. Research on glacier surface ice algae, and on cyanobacterial mats which grow in Antarctic melt streams, has shown that organisms produce pigments which protect them from UV (McKnight *et al.*^1999^; Remias *et al.*^2012^; Yallop *et al.*^2012^; Foreman *et al.*^2013^). However, there has been little investigation of the degree of light stress and adaptation mechanisms operating in cryoconite hole and cryolake communities, which are likely dominated by different species to the bare ice surfaces (Takeuchi 2013). One study of cryoconite communities from the Blue Ice ecosystem near Vestfold Hills demonstrates that they are likely low light adapted during short-term incubations (Hodson *et al.*^2013^). Our data extend this work by prolonging the incubation period and investigating the photophysiology of the community as a whole.

The field measurements demonstrate that the ice lid severely limits the proportion of solar radiation that can reach the sediment layer in larger Antarctic cryoconite holes and cryolakes. The proportion of PAR transmitted to the base of the cryolake is, on average, <10% of that received on the ice surface, primarily as a result of reflection and scattering (Warren, Brandt and Grenfell 2006). This is a similar scenario to that found in the perennially ice-covered lakes of the Dry Valleys. PAR attenuation at 10 m depth in these oligotrophic water bodies beneath the ice cover is up to 99% (Fritsen and Priscu 1999), and so the photosynthetic organisms which inhabit the lake must adapt to extremely low light levels (Morgan-Kiss *et al.*^2006^) to maximize the photosynthetic efficiency, for example, by locating large numbers of chlorophyll pigments at each photosynthetic center (Lizotte and Priscu 1992).

There are diurnal and seasonal changes in PAR penetration within cryoconite hole and cryolake habitats associated with the melting of the ice lids. As the ablation season progresses, an increasing percentage of surface PAR is transmitted to the debris layers where the majority of microorganisms are located. This is illustrated in Fig. 2. Once internal melting of the cryolake has occurred, indicated by the spike in conductivity and increase in water temperature at day 20, the PAR attenuation decreases dramatically and clear diurnal cycles in PAR are detected at the sediment layer between day 20 and 26. On day 27, a freezing event decreases the water temperature and increases the PAR attenuation. Daily PAR maxima at the base of the Canada Glacier cryoconite hole rose from 150 to 400 μmol m^2^ s^−1^ following the melt event on 22 January (Fig. 4), and daily maximum PAR attenuation in the cryolake decreased from 85% to 75% during the same event (Fig. 2). This was primarily a result of the ice lid thinning, likely melting from below. The PAR attenuation recorded in the cryoconite hole was similar to that observed by Hodson *et al.* (2013) in cryoconite holes in the Blue Ice areas of the Vestfold Hills. Melting of the ice not only causes thinning of the ice cover, but changes the optical properties, with the ice appearing more bubbly and ‘whiter’ as the season progress. This phenomenon is also observed in adjacent ice-covered lakes (Fritsen and Priscu 1999), although PAR propagation through the glacier ice lid is generally higher than through the lake ice caps (Hodson *et al.*^2013^). Ice at the top of the lid is opaque and bubbly because of rapid freezing, and hence exhibits very high attenuation. In contrast, the ice at the base of the lid is formed by slow refreezing of the meltwater below, during which bubbles are expelled. This reduces reflection and increases scattering (Warren, Brandt and Grenfell 2006), and so more radiation reaches the base of the cryolake as the season progresses (Figs 2 and 4). Our measurements demonstrate that water attenuates comparatively little of the incoming PAR, since small changes in water depth had little effect on PAR measured at the sediment layer.

PAR receipt at the sediment layer was an important regulator of productivity in the experimental microbial communities. The time taken for the incubations to reach a state of net P is controlled by light, temperature and availability of carbon and other nutrients. Previous experiments have demonstrated that the communities are capable of recycling nutrients and employ strategies to extract N and P from minerals in the cryoconite material (including nitrogenase and phosphatase) (Stibal *et al.*^2008^; Telling *et al.*^2011^; Segawa *et al.*^2014^). The availability of dissolved inorganic carbon is controlled by heterotrophic activity in the sediment layer and dissolution of CaCO_3_ from cryoconite (Bagshaw *et al.*^2016^), with comparatively little introduced by melting of ice below the sediment layer. Since the sediment layer depth was identical in all our incubations, there should be little variation in carbon and nutrient availability between experiments, hence the primary controls were temperature and light intensity. In the low-temperature incubations (where PAR ranged from 15 to 60 μmol m^2^ s^−1^), higher PAR resulted in more efficient photosynthetic communities, with the incubations reaching a state of net P more rapidly when light intensity increased. However, when light intensity was further increased from 74 to 275 μmol m^2^ s^−1^, the communities became less efficient.

Filamentous cyanobacteria can adjust the length of light-harvesting antennae according to the level of available irradiance (Huner, Oquist and Sarhan 1998; Morgan-Kiss *et al.*^2006^). Research in the Arctic has suggested that photosynthetic cells are located on the outside of cryoconite granules to obtain the most light (Langford *et al.*^2010^). We therefore anticipate that adaptations to low light are operating in the cryoconite hole and cryolake communities. Analysis of fluorescence data indicated that both the Antarctic and Arctic communities were adapted to low light and showed no obvious capability to acclimate between light levels. If light acclimation had been observed, rETR_max_ would have increased and α decreased in high light and vice versa in low light, as cells acclimate to maximize electron transport rate under higher light whilst investing less effort in maximizing light use efficiency (Horton, Ruban and Walters 1996). As a result, the light saturation coefficient E_k_ would increase under high light as cells acclimate to utilize the greater photodose available. However, in this study, data for rETR_max_, α and E_k_ showed the same patterns, with significant correlation between rETR_max_ and α. All three parameters increased under low light and decreased at higher light, independent of the order of exposure, e.g. on transfer from high to low and low to high light the same changes were observed. This demonstrates a lack of photoacclimation to high light and suggests that cells were adapted to low light and had no capacity to change their photochemistry over the duration of each experimental incubation.

NPQ increased as a function of PAR for all light curves, both for Arctic and Antarctic samples transferred from both high to low and low to high light, and NPQ did not saturate by the end of the light curve. A lack of saturation in NPQ would suggest that, despite the photophysiological stress induced under high light, there was still physiological capacity in the form of photoprotective downregulation by, for example, inducing xanthophyll cycling to prevent photodamage (Ting and Owens 1993; Lavaud, Rousseau and Etienne 2002; Lavaud and Kroth 2006). This would explain the ability of samples to respond to the transfer from high light to low light and show a recovery through the observed increases in rETR_max_ and α. Interestingly, the Arctic samples exhibited similar fluorescence results to the Antarctic samples. We had hypothesized that the Arctic communities would be better adapted to cope with high radiation, since the ice lids of cryoconite holes and supraglacial lakes situated there tend to melt out in the summer months. However, the communities displayed similar rETR_max_ to the Antarctic experiments, and were similarly significantly less efficient at high light levels. In general, data for NPQ indicated light stress at high light in both experiments, although Antarctic samples appeared to have a significantly greater ability to induce NPQ when not stressed (i.e. at low light) compared to Arctic samples. Both sample sets demonstrated that NPQ may occur, in agreement with data on ice algae presented by Yallop *et al.* (2012).

The key difference between the Arctic and Antarctic communities was that the Arctic communities did not reach a state of net P; P and R remained in balance throughout the incubation. This is in agreement with several *in situ* and laboratory studies of cryoconite production rates (Stibal and Tranter 2007; Telling *et al.*^2010^; Hodson *et al.*^2013^). We speculate that the Arctic communities are adapted to frequent redistribution by meltwater, and so form microcommunities within the cryoconite which are bound together for long periods (Cook *et al.*^2015^). In contrast to the Antarctic system, which rely on diffusion of dissolved inorganic carbon from sediment layers (Bagshaw *et al.*^2016^), the Arctic communities form tightly knit granules where phototrophic and heterotrophic processes occur in close proximity. The formation of granules may also play a role in protecting Arctic communities from light stress, by providing intermittent shading as the granules move, and by extracellular polymeric substance shielding (Langford *et al.*^2014^).

Polar glacier surfaces are exposed to extremely high light levels for several months per year, which can cause significant stress to microorganisms. Nevertheless, since the growing season is short, they must utilize this light when it is available. Our experiments demonstrate that PAR is a controlling factor on cryoconite and cryolake production. Production is limited by low PAR; and conversely, by very high PAR. Antarctic cryoconite and cryolake dwelling phototrophs are therefore well adapted to ‘Goldilocks’ light conditions: not too much, not too little. When communities are covered by an ice lid, organisms are most suited to low light environments and are stressed by high light. When communities are rarely ice covered during the summer months, as in many Arctic systems, they must develop strategies to protect against high PAR and UV stress. These include secondary pigmentation (Remias *et al.*^2012^; Yallop *et al.*^2012^), which can also protect against freeze–thaw processes (Dieser, Greenwood and Foreman 2010), and formation of extracellular polymeric substance-bound granules (Langford *et al.*^2014^). We speculate that such granules are rarely observed in Antarctic cryoconite holes and cryolakes because the organisms are (a) in a more hydrologically stable environment with limited mixing (Bagshaw *et al.*^2010^) and (b) usually covered by an ice lid which attenuates UV radiation.

## CONCLUSIONS

Measurements of physical, chemical and biological parameters in polar cryoconite and cryolake ecosystems revealed that light and temperature are key controls on primary production. Melting of ice lids prompts both physical and chemical changes, caused by temperature fluctuation and increased penetration of light to microorganisms concentrated in sediment layers. Receipt of PAR is a major control on primary production efficiency in cryoconite and cryolake ecosystems. However, more light does not necessarily result in higher production. Microbial communities in Antarctic cryolakes which are usually covered by an ice lid are more efficient at moderate light levels; production decreases when light intensity is increased to levels nearing that measured at the glacier surface. Measurements of photophysiology demonstrate that the communities are stressed when exposed to high light and do not acclimate, although they adopt strategies to protect against photodamage. Interestingly, Arctic cryoconite communities were also stressed at high light levels, so we speculate that adaptations such as pigmentation, granule formation and photophysiological adjustment are important for maintaining ecosystem productivity throughout the melt season.
